# Dystrophy of Oligodendrocytes and Adjacent Microglia in Prefrontal Gray Matter in Schizophrenia

**DOI:** 10.3389/fpsyt.2020.00204

**Published:** 2020-03-26

**Authors:** Natalya A. Uranova, Olga V. Vikhreva, Valentina I. Rakhmanova, Diana D. Orlovskaya

**Affiliations:** Laboratory of Clinical Neuropathology, Mental Health Research Center, Moscow, Russia

**Keywords:** prefrontal cortex, postmortem, oligodendrocytes, microglia, ultrastructure, morphometry, schizophrenia

## Abstract

**Background:**

Some evidence support the notion that microglia activation in acute state of schizophrenia might contribute to damage of oligodendrocytes and myelinated fibers. Previously we found dystrophic changes of oligodendrocytes in prefrontal white matter in schizophrenia subjects displaying predominantly positive symptoms as compared to controls. The aim of the study was to verify whether microglial activation might contribute to dystrophic changes of oligodendrocytes in prefrontal gray matter in this clinical subgroup.

**Methods:**

Transmission electron microscopy and morphometry of microglia and adjacent oligodendrocytes were performed in layer 5 of the prefrontal cortex (BA10) in the schizophrenia subjects displaying predominantly positive symptoms (SPPS, n = 12), predominantly negative symptoms (SPNS, n = 9) and healthy controls (n = 20).

**Results:**

Qualitative study showed microglial activation and dystrophic alterations of microglia and oligodendrocytes adjacent to each other in both subgroups as compared to controls. A significant reduction in volume density (Vv) and the number (N) of mitochondria and an increase in N of lipofuscin granules were found in oligodendrocytes and adjacent microglia in both subgroups. Vv of lipofuscin granules, Vv and N of vacuoles of endoplasmic reticulum in microglia were increased significantly in the SPPS subgroup as compared to controls. In the SPPS subgroup Vv and N of mitochondria in microglia were correlated with N of vacuoles in microglia (r = -0.61, p < 0.05) and with Vv (r = 0.79, p < 0.01) and N (r = 0.59, p < 0.05) of mitochondria in oligodendrocytes. Vv of mitochondria in microglia was also correlated with Vv and N of vacuoles in oligodendrocytes in the SPPS subgroup (r = 0.76, p < 0.01). Area of nucleus of microglial cells was correlated negatively with age (r = -0.76, p < 0.01) and age at illness onset (r = -0.65, p < 0.05) in the SPPS subgroup. In the SPNS subgroup N of mitochondria in microglia was correlated with Vv of lipofuscin granules in oligodendrocytes (r = -0.9, p < 0.01). There were no significant correlations between these parameters in the control group.

**Discussion:**

Microglial dystrophy might contribute to oligodendrocyte dystrophy in the schizophrenia subjects with predominantly positive symptoms during relapse. Mitochondria in microglia and oligodendrocytes may be a target for treatment strategy of schizophrenia.

## Introduction

Neuroimaging studies provide increasing evidence for functional dysconnectivity and the alteration of white matter integrity in schizophrenia (1, 2). The prefrontal cortex (PFC) is one of the main brain structure affected in schizophrenia (3). Functional dysconnectivity of the PFC is known to be associated with psychotic symptoms and cognitive dysfunctions in schizophrenia (4). Severity of positive symptoms is linked with microstructural abnormalities in the PFC (5) and functional dysconnectivity in fronto-temporal cortex in patients with schizophrenia (6). Garver et al. (7) in DTI study showed that an increase in Dm in drug-free schizophrenia patients was reduced following the reduction of psychotic symptoms after antipsychotic drug treatment.

Pathology of oligodendrocytes and myelin is considered to be a biological basis for dysconnectivity in schizophrenia (2, 8). Reduced oligodendrocyte density (9–12), ultrastructural alterations of oligodendrocytes and myelinated fibers (13–15), altered intracortical myelin staining (16) and impaired differentiation of oligodendrocyte precursors (17) have been reported in the PFC in schizophrenia. Abnormalities of myelination, reduced expression and dysregulation of oligodendrocyte and myelin-related genes (18–20) have been detected in the PFC in schizophrenia.

The mechanisms of oligodendrocyte and myelin abnormalities in schizophrenia remain uncertain. Oligodendrocytes are often located adjacent to microglial cells in both control and schizophrenia (21). Previously we found dystrophic alterations in oligodendrocytes adjacent to microglia and myelin in the prefrontal white matter in the schizophrenia patients with predominantly positive symptoms as compared to controls (21). These data suggest the involvement of microglial activation in oligodendrocyte abnormalities in schizophrenia. Microglia hypothesis of schizophrenia supposes that microglial overactivation is crucial in the etiology of schizophrenia (22). Multiple lines of evidence for a role of neuroinflammation and oxidative stress in the pathophysiology of psychosis (23) and increased expression of genes associated to immune and chaperone functions in the PFC (24) have been reported in schizophrenia. Microglial activation in brain of the schizophrenia patients has been reported both *in vivo* (25) and postmortem (26, 27) studies. Some postmortem studies detected an increased microglial density in a subpopulation of schizophrenia patients (28–32). Steiner et al. (32) showed a significantly increased density of activated microglia in patients with schizophrenia who committed suicide during acute psychosis as compared to nonsuicidal patients with schizophrenia in the dorsolateral PFC. Busse et al. (33) found a significant difference in microglial density between patients with paranoid and residual schizophrenia. Fillman et al. (31) have reported increased inflammatory mRNA expression in ~40% of schizophrenia patients. These data indicate increased microglial density in a subpopulation of patients with schizophrenia.

Neuroimaging studies of psychotic patients support a link between microglial activation and psychosis (34), sub-clinical psychotic symptoms (35), severity of psychotic symptoms (36), age- dependent pattern of microglial activation in male psychosis patients (37), illness duration (38). Fillman et al. (39) have recently reported that schizophrenia patients with increased cytokine level showed significantly worse than patients with low-cytokine level on verbal fluency and reduced volume of pars opercularis (Broca's area). Dysregulated glutathione homeostasis was found in the PFC in early psychosis patients (40). Patients with early onset of schizophrenia showed greater and progressive loss of frontal gray matter in the first few years after disease onset. (41). These data suggest that neuroinflammation might be present in different subgroups of schizophrenia patients. Previously we found lowered oligodendrocyte density in layer 5 of the PFC in the schizophrenia patients (12). We hypothesized that oligodendrocyte dystrophy may be at least in part associated with increased activation of microglial cells located adjacent to dystrophic oligodendrocytes in the schizophrenia patients during acute psychosis. We aimed to estimate ultrastructural parameters of microglia and oligodendrocytes adjacent to each other in layer 5 of the PFC in two subgroups of patients with schizophrenia - those displaying predominantly positive symptoms and those displaying predominantly negative symptoms.

## Material and Methods

### Subjects

21 cases with schizophrenia and 20 normal controls were studied. Postmortem brain tissue was obtained from the Anatomical Department of Moscow Psychiatric Hospitals №1 and № 15 and Moscow Higher Medical School. ICD-10 and DSM-IV-R diagnostic criteria were used by psychiatrists. Independent psychiatrists examined medical records using the Scale for the Assessment of Negative Symptoms (SANS) and the Scale for the Assessment of Positive Symptoms (SAPS) to rate negative and positive symptoms during the last hospitalization in the schizophrenia patients. The predominantly positive or the predominantly negative symptoms were estimated based on some integrative characteristics including common score of positive and negative symptoms (in points), relative frequency (%) of positive and negative symptoms, and relative frequency (%) of the most severe positive and negative symptoms. Basic demographic and clinical data are given in **Table 1**. Consent for autopsy was obtained and approval for the study from the Ethics Committee of Mental Health Research Center was received. Exclusion criteria were alcohol, drug abuse, other neuropsychiatric disorders. Cases were excluded from the study by an experienced neuropathologist if there was evidence for neurological damage, neoplasmic, vascular and neurodegenerative changes. Cases were coded and morphometric study was performed blindly. Samples of the frontal lobe (Brodmann's area 10) from the left hemisphere were dissected from the brains. Data on age at onset, duration of illness and neuroleptic exposure were obtained from medical records. Also chlorpromazine equivalents were estimated for the patient's last 30 days. Causes of death were the same in the control and the schizophrenia groups [see (21)].

**Table 1:** Demographic and clinical data (mean±standard deviation).

Subjects	Number per group	Gender^a^	Age (years)^b^	PMI (hours)^c^	Duration of illness (years)	NTR (chlorpromazine equivalents)
			Mean±SD	Mean±SD	Mean±SD	Mean±SD
Controls	20	12M, 8F	58.3±12.6	6.1±1.0		
Schizophrenia	21	11 M, 10F	56.3±16.8	6.2±1.0	28.2±13.0	406.5±303.6
SPNS	9	5M, 4F	56.2±17.8	5.6±0.7	29.3±15.7	457.0±378.6
SPPS	12	6M, 6F	56.4±16.8	6.6±1.1	27.42±13.0	367.2±253.1

PMI, postmortem interval. SPPS, the subgroup with predominantly positive symptoms; SPNS, the subgroup with predominantly negative symptoms; NTR, neuroleptic treatment. Controls vs. SCZ: ^a^Chi-square test (p = 0.43), ^b^ANOVA (p = 0.92), ^c^ANOVA (p = 0.24).

### Tissue Preparation

Tissue preparation was described in details in our previous paper (21). Sections were viewed with a JEM-100B (JEOL, Japan) electron microscope. Electron micrographs of oligodendrocytes were obtained at *3300 magnification. Oligodendrocytes were identified by small round or oval nucleus, short cisternae of granular endoplasmic reticulum, polyribosomes and the Golgi complex. Microglial cell bodies had elongated, round or triangular nuclei with electron- dense heterochromatin. Cytoplasm of these cells contains mitochondria, cisternae of endoplasmic reticulum, lysosomes, lipofuscin granules, Golgi complex.

### Morphometry

Morphometry was performed for microglia adjacent to oligodendrocytes and oligodendrocytes adjacent to microglia. Mean number of microglia/oligodendrocyte pairs per case ± S.D. collected were: 11.8 ± 3.5 for the control group and 13.05 ± 3.6 for the schizophrenia group. These cells were measured within the area (mean ± S.D.) 0.21 ± 0.06 mm^2^ in the control group and 0.19 ± 0.05 mm^2^ in the schizophrenia group. Cell density was estimated as the number of cells per unit tissue area (N/mm^2^). Cell size, nucleus/cytoplasm ratio, volume fraction (Vv) and the number (N) of organelles (mitochondria, vacuoles of endoplasmic reticulum, lipofuscin granules) were measured. Areas of cells and cellular organelles were estimated using test grids for two-dimensional counts, superimposed on the negatives at the final magnification *26,000. The counting method was previously described in details (21).

### Statistical Analysis

Statistica software, version 7 (Stat Soft) was used for statistical analysis. The data were examined using the Kolmogorov-Smirnov test for normality. A Pearson correlation analysis was performed to detect possible correlations between the parameters measured and age, postmortem interval, treatment with antipsychotic drugs (CPZ equivalents), illness duration. The groups did not differ significantly by age (p = 0.9) and postmortem delay (p = 0.2). ANCOVA comparisons between the schizophrenia patients and controls were performed with cell parameters measured as dependent variables, diagnosis as independent factor, and age and post-mortem interval as covariates. To determine the effect of clinical subgroups on the parameters measured, ANCOVA was used with cell parameters as dependent variables, clinical SPPS and SPNS subgroups as between-subjects factors, and age and postmortem interval as covariates. To determine the effect of gender, duration of disease (< 26 years and >26 years, group median), age at onset of disease on the parameters measured ANCOVA was used with the cell parameters as dependent variables, diagnosis and gender or disease duration or age at onset of disease as between-subjects factors, and age and postmortem interval as covariates. Following ANCOVA, we performed a post-hoc Duncan test. Cohen's d in the Statistica software was used to determine effect size.

## Results

**Figure 1:**
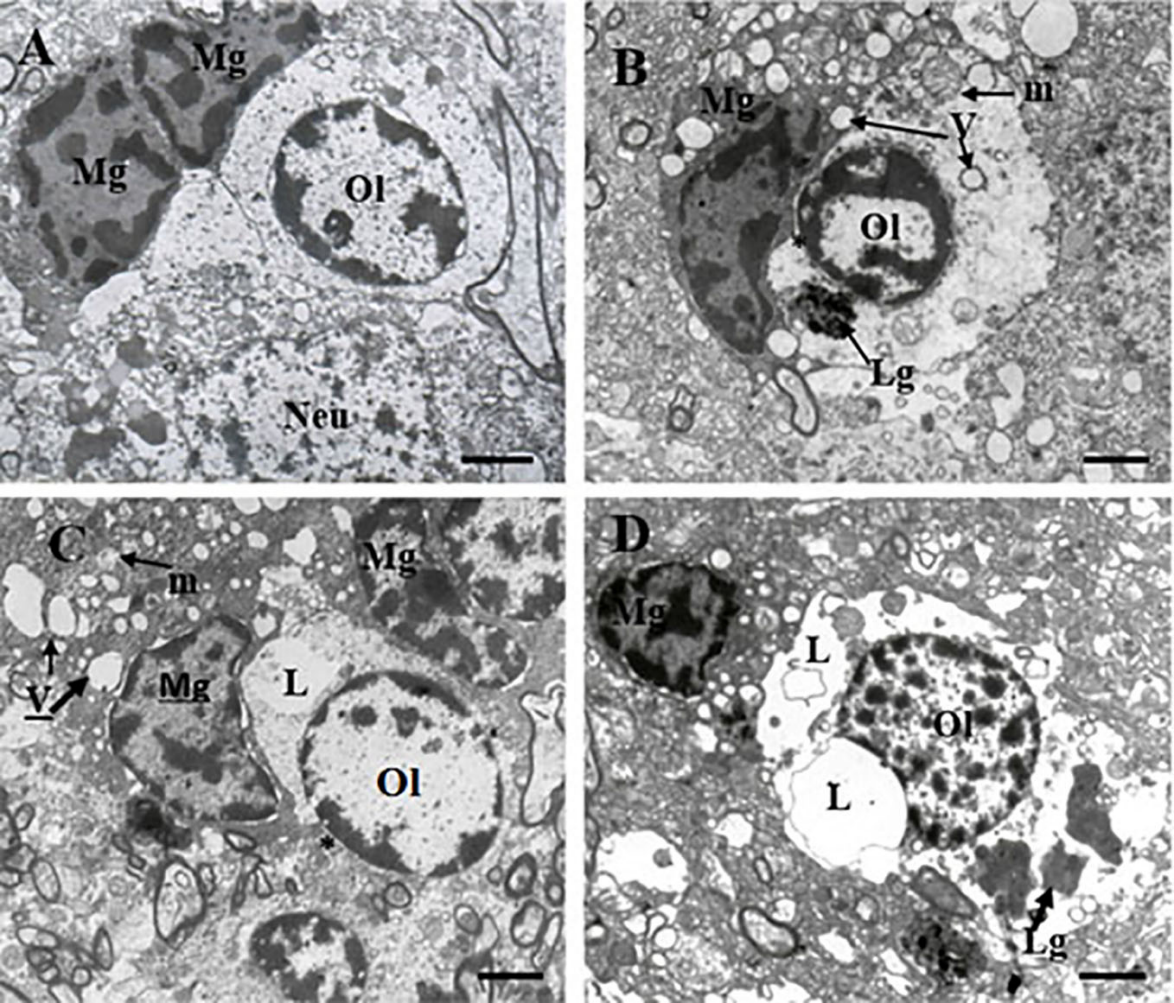
These micrographs from layer 5 of the prefrontal cortex show microglia adjacent to oligodendrocytes from control brain **(A)** and from the SPPS subgroup **(B–D)**. “Resting” microglia **(A)**. Ameboid (activated) microglia **(B)**. Dystrophic changes in microglia and oligodendrocytes: cytoplasm vacuolation, damaged mitochondria, accumulation of lipofuscin granules **(C, D)**. Microglial cytoplasm contacts with oligodendrocyte nucleus (**B**, **C**, *). Focal lysis (L) of cytoplasm of oligodendrocytes adjacent to microglia **(C, D)**. Mg, microglia; Ol, oligodendrocyte, m, mitochondria (arrows); V, vacuole (arrows), Lg, lipofuscin granule (arrows). (Scale bars = 1 µm).

**Figure 2:**
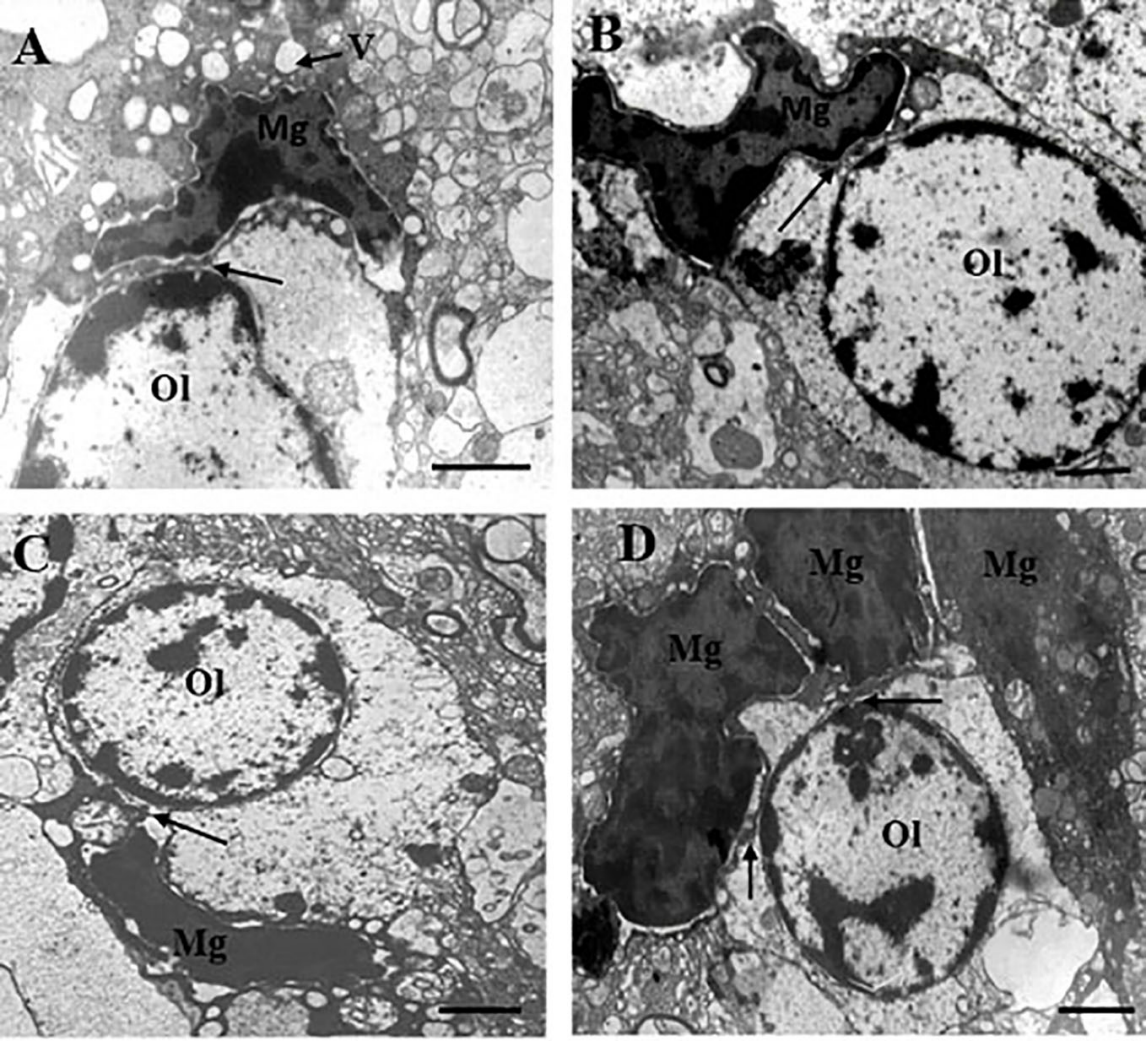
These micrographs from layer 5 of the prefrontal cortex show microglia adjacent to oligodendrocytes from the SPNS subgroup. Dark dystrophic microglia **(A)**. Ameboid microglia **(B)**, signs of apoptosis (dark nucleus and small rim of dark shrunken cytoplasm) **(C, D)**. All microglia showed contacts with the nuclei of oligodendrocytes (**A–D**, arrows). A group (cluster) of three microglia surrounding dystrophic oligodendrocyte **(D)**. Ol, oligodendrocyte; Mg, microglia. Scale bar=1 µm.

**Table 1** contains demographic and clinical data. The ultrastructure of microglia was heterogeneous in the control group and in both clinical subgroups of schizophrenia subjects. Four different ultrastructural types of microglia were found: “resting”, ameboid (activated), dystrophic and apoptotic. “Resting” microglia contained relatively small cytoplasm, few mitochondria and cisterns of endoplasmic reticulum (**Figure 1A**). Ameboid (activated) microglial cells was detected by cytoplasmic hypertrophy, many normal mitochondria and vacuoles of enlarged cisterns of endoplasmic reticulum. (**Figure 1B**). Dystrophic microglia contained few swollen mitochondria with destroyed cristae, numerous vacuoles of enlarged cisterns of endoplasmic reticulum of different size and lipofuscin granules (**Figures 1C, D**). The nucleus of many dystrophic microglial cells was dark due to electron-dense cytoplasm and nucleoplasm (**Figure 2A**). Some of these cells showed signs of apoptosis (dark nucleus and small rim of dark shrunken cytoplasm (**Figures 2B–D**). Activated (**Figure 1B**), dystrophic and apoptotic microglia (**Figures 2A–D**) were often rod-shaped and touched the nucleus of oligodendrocytes suggesting direct contacts. Intermediate subtypes of microglia were also observed in both subgroups studied and in the control group. Oligodendrocytes adjacent to microglia in schizophrenia cases looked swollen, vacuolated as compared to controls, contained small amount of ribosomes and lipofuscin granules (**Figure 1C**). Some oligodendrocytes mostly in the schizophrenia subjects contained abnormal large vacuoles, a sign of focal lysis of their cytoplasm (**Figures 1B, C**).

We found the effect of diagnosis on the parameters of microglia adjacent to oligodendrocytes (**Table 2**). Schizophrenia group showed a significant decrease in area, Vv and N of mitochondria (p < 0.001) and a significant increase in area, N (p < 0.05) and Vv of vacuoles (p < 0.01) as well as an increase in area, N (p < 0.01) and Vv (p < 0.001) of lipofuscin granules as compared to the control group. A significant effect of diagnosis was also found on Vv and N of mitochondria, vacuoles, and lipofuscin granules in oligodendrocytes (**Table 2**). Area, Vv and N of mitochondria (p < 0.001) were lower but area (p < 0.01) and N (p < 0.05) of vacuoles, area, Vv and N of lipofuscin granules (p < 0.001), area of oligodendrocyte cell and cytoplasm (p < 0.01) were higher in the schizophrenia group as compared to the control group. Effect size for these parameters of microglia and adjacent oligodendrocytes was large (**Table 2**). Cell density was not changed (**Table 2**).

**Table 2:** Effect of diagnosis on microglia adjacent to oligodendrocytes and on oligodendrocytes adjacent to microglia.

	Microglia adjacent to oligodendrocytes											
	Controls (n=20)	Schizophrenia (n=21)	F(1,37)	p	Cohen’s d	Controls (n=20)	SPPS (n=12)	SPNS (n=9)	F(2,36)	p	Cohen’s d	
	Mean±SEM	Mean±SEM				Mean±SEM	Mean±SEM	Mean±SEM			SPPS/ Controls	SPNS/ Controls
Areaofmicroglia(μm^2^)	18.92±0.01	19.61±0.9	0.26	0.615	0.20	18.92±0.01	18.95±0.9	20.49±1.7	0.88	0.424	0,01	0.43
Areaofnucleus (μm^2^)	10.14±3.6	10.19±0.5	<0.00	0.985	0.03	10.14±3.6	10.01±0.4	10.43±1.0	0.04	0.956	0,09	0.15
Areaofcytoplasm(μm^2^)	8.78±0.3	9.42±0.7	0.42	0.522	0.24	8.78±0.3	8.93±0.7	10.07±1.3	1.19	0.315	0,07	0.45
Nucleus/cytoplasmratio	1.95±1.7	1.95±0.2	0.01	0.913	0.01	1.95±1.7	1.95±0.3	1.96±0.3	0.15	0.863	0,00	0.02
Areaofmitochondria (μm^2^)	0.90±1.0	0.38±0.01	29.47	<0.001***	**1.33**	0.90±1.0	0.35±0.1	0.40±0.1	14.56	<0.001***	**1,34**	**1.27**
Vvofmitochondria (%)	10.15±1.3	3.77±0.5	50.98	<0.001***	**1.50**	10.15±1.3	3.37±0.8	4.31±0.7	25.33	<0.001***	**1,53**	**1.49**
N ofmitochondria	1.68±0.2	0.65±0.1	33.91	<0.001***	**1.36**	1.68±0.2	0.54±0.1	0.80±0.2	17.60	<0.001***	**1,44**	**1.23**
Areaofvacuoles (μm^2^)	0.85±0.7	1.57±0.2	7.27	<0.05*	**0.77**	0.85±0.7	1.69±0.3	1.41±0.3	4.11	<0.05*	**0,88**	0.76
Vvofvacuoles (%)	9.25±0.01	17.54±2.5	9.79	<0.01**	**0.83**	9.25±0.01	19.51±3.7	14.91±3.2	6.35	<0.01**	**1,01**	0.77
N ofvacuoles	1.70±0.1	3.20±0.5	6.19	<0.05*	**0.72**	1.70±0.1	3.48±0.8	2.81±0.7	3.75	<0.05*	**0,84**	0.67
Area of lipofuscin granules μm^2^)	0.21±0.1	0.56±0.1	11.96	<0.01**	**1.09**	0.21±0.1	0.61±0.1	0.49±0.1	5.81	<0.01**	**1,28**	**1.08**
Vvoflipofuscingranules (%)	1.80±1.0	5.28±0.9	13.10	<0.001***	**0.99**	1.8±1.0	6.03±1.3	4.29±1.0	7.01	<0.01**	**1,15**	**0.99**
N oflipofuscingranules	0.11±0.2	0.32±0.1	9.95	<0.01**	**0.90**	0.11±0.2	0.30±0.1	0.33±0.1	5.37	<0.01**	**1,06**	**0.99**
Cell density (N /mm^2^)	59.97±4.1	69.88±5.4	1.50	0.228	0.42	59.97±4.1	70.04±8.8	69.67±5.3	0.75	0.478	0,40	0.48
Oligodendrocytes adjacent to microglia												
Areaofoligodendrocyte(μm^2^)	38.87±0.7	45.97±1.8	9.34	<0.01**	**0.87**	38.87±0.7	43.84±2.0	48.8±3.1	5.50	<0.01**	0,71	**1.15**
Areaofnucleus (μm^2^)	15.67±5.3	18.4±1.0	3.79	0.059	0.60	15.67±5.3	17.25±1.3	19.93±1.6	2.60	0.088	0,38	**0.91**
Areaofcytoplasm (μm^2^)	23.21±0.01	27.57±1.1	8.75	<0.01**	**0.85**	23.21±0.01	26.6±0.9	28.87±2.2	4.66	<0.05*	0,79	**0.99**
Nucleus/cytoplasmratio	0.81±2.0	0.78±0.01	0.26	0.614	0.15	0.81±2.0	0.77±0.1	0.80±0.1	0.23	0.794	0,21	0.05
Areaofmitochondria(μm^2^)	1.31±0.1	0.75±0.1	30.53	<0.001***	**1.26**	1.31±0.1	0.76±0.1	0.74±0.1	15.10	<0.001***	**1,23**	**1.35**
Vvofmitochondria (%)	5.88±3.1	2.75±0.3	42.67	<0.001***	**1.39**	5.88±3.1	2.9±0.5	2.56±0.3	20.84	<0.001***	**1,31**	**1.48**
N ofmitochondria	2.70±1.6	1.52±0.1	35.45	<0.001***	**1.31**	2.70±1.6	1.53±0.2	1.50±0.2	17.52	<0.001***	**1,27**	**1.43**
Areaofvacuoles (μm^2^)	0.28±2.2	0.84±0.2	8.83	<0.01**	**1.02**	0.28±2.2	0.83±0.3	0.84±0.2	4.24	<0.05*	**1,06**	**1.06**
Vvofvacuoles (%)	0.80±0.2	2.01±0.5	3.64	0.065	0.64	0.80±0.2	1.66±0.7	2.47±0.7	2.81	0.074	0,45	**0.95**
N ofvacuoles	0.32±0.3	0.84±0.2	4.17	<0.05*	0.65	0.32±0.3	0.48±0.2	1.31±0.4	7.09	<0.01**	0,34	**1.13**
Area of lipofuscin granules (μm^2^)	0.32±0.01	0.91±0.1	28.62	<0.001***	**1.33**	0.32±0.01	0.94±0.1	0.88±0.1	14.06	<0.001***	**1,35**	**1.59**
Vvoflipofuscingranules (%)	1.18±0.1	3.43±0.4	30.51	<0.001***	**1.32**	1.18±0.1	3.62±0.6	3.17±0.4	15.45	<0.001***	**1,38**	**1.55**
N oflipofuscingranules	0.21±0.1	0.63±0.1	33.22	<0.001***	**1.37**	0.21±0.1	0.68±0.1	0.54±0.1	18.37	<0.001***	**1,45**	**1.57**
Cell density (N /mm^2^)	59.04±4.4	67.75±5.2	1.25	0.271	0.38	59.04±4.4	66.42±8.5	69.53±5.0	0.73	0.487	0,30	0.54

*p < 0.05, **p < 0.01, ***p < 0.001.

**Figure 3:**
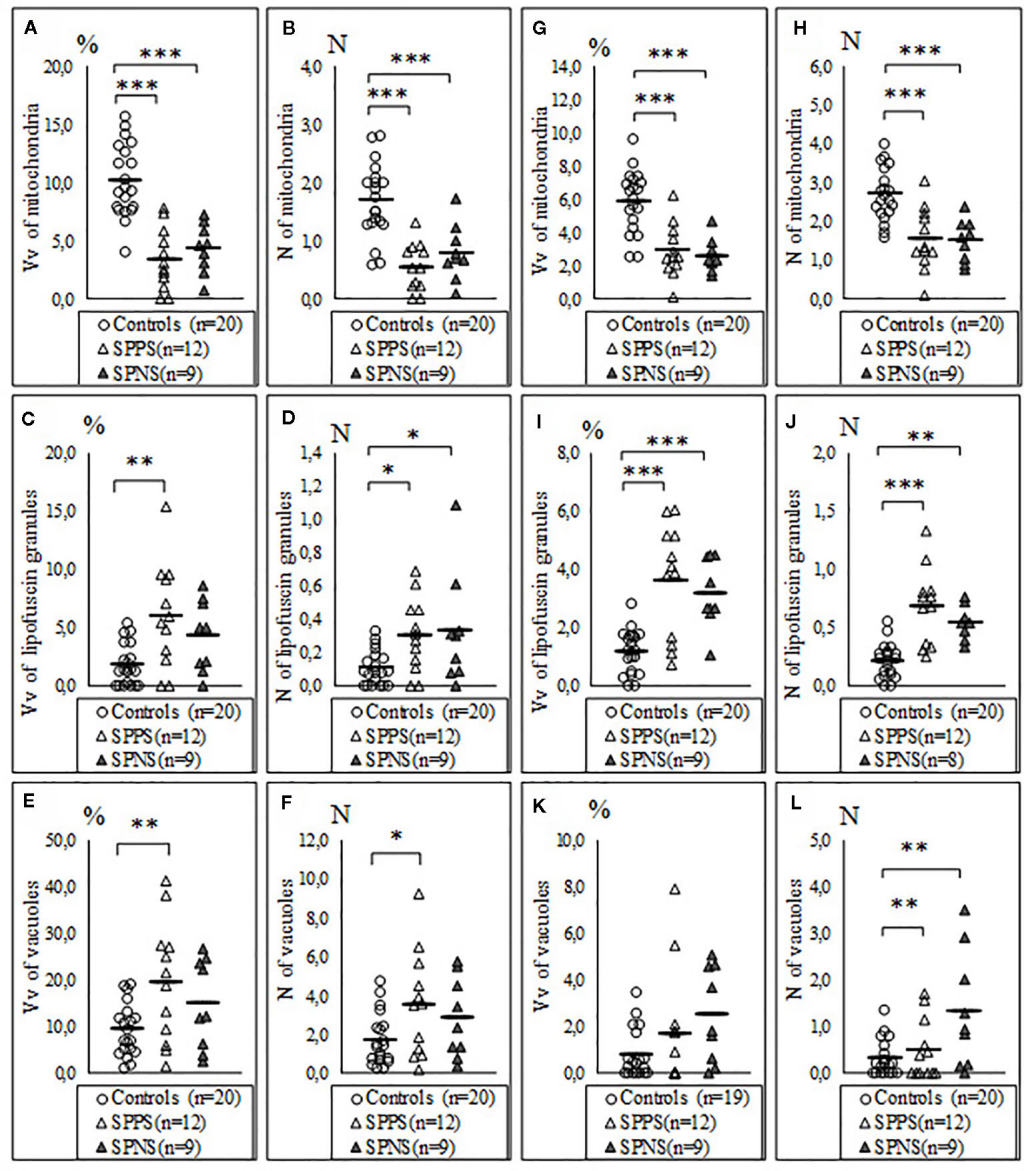
Individual and mean values for Vv and N of mitochondria **(A, B)**, lipofuscin granules **(C, D)**, vacuoles **(E, F)** in microglia adjacent to oligodendrocytes, in oligodendrocytes adjacent to microglia **(G–L)** for the control group, the subgroup with predominantly positive symptoms (SPPS) and the subgroup with predominantly negative symptoms (SPNS). *p < 0.05, **p < 0.01, ***p < 0.001.

We also found a significant effect of clinical subgroups on the same ultrastructural parameters of microglia and oligodendrocytes adjacent to each other (**Table 2**). Post-hoc showed a significantly lowered Vv and N of mitochondria in the SPPS subgroup (p < 0.01) and in the SPNS subgroup (p < 0.001) in both microglia (**Figures 3A, B**) and in oligodendrocytes (**Figures 3G, H**) as compared to controls. Vv of lipofuscin granules in microglia was increased only in the SPPS subgroup (p < 0.01), but N of lipofuscin granules in microglia, Vv and N of lipofuscin granules in oligodendrocytes were higher in both clinical subgroups as compared to controls (**Figures 3B, I, J**). Vv and N of vacuoles in microglia were higher only in the SPPS subgroup (p < 0.01, p < 0.05) compared to controls (**Figures 3C–F**). However, in oligodendrocytes Vv of vacuoles was not changed significantly, and N of vacuoles were higher in both clinical subgroups (p < 0.01) (**Figures 3K, L**).

**Figure 4:**
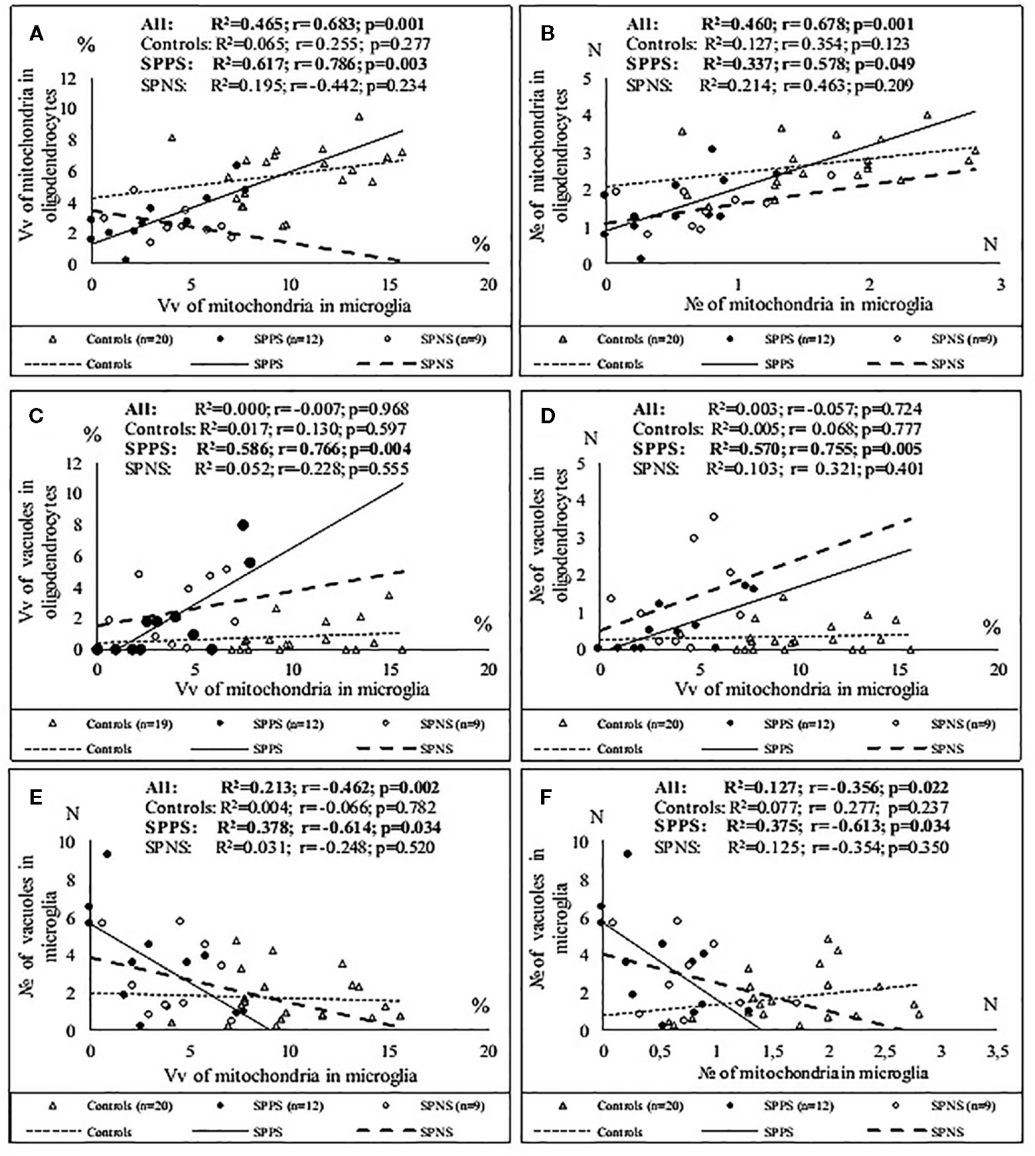
Positive significant correlations between Vv and N of mitochondria in microglia and Vv and N of mitochondria in oligodendrocytes **(A, B)**; positive significant correlations between Vv of mitochondria in microglia and Vv and N of vacuoles in oligodendrocytes **(C, D)** and negative significant correlations between Vv and N of mitochondria and N of vacuoles in microglia **(E, F)** in the SPPS subgroup.

**Figure 5:**
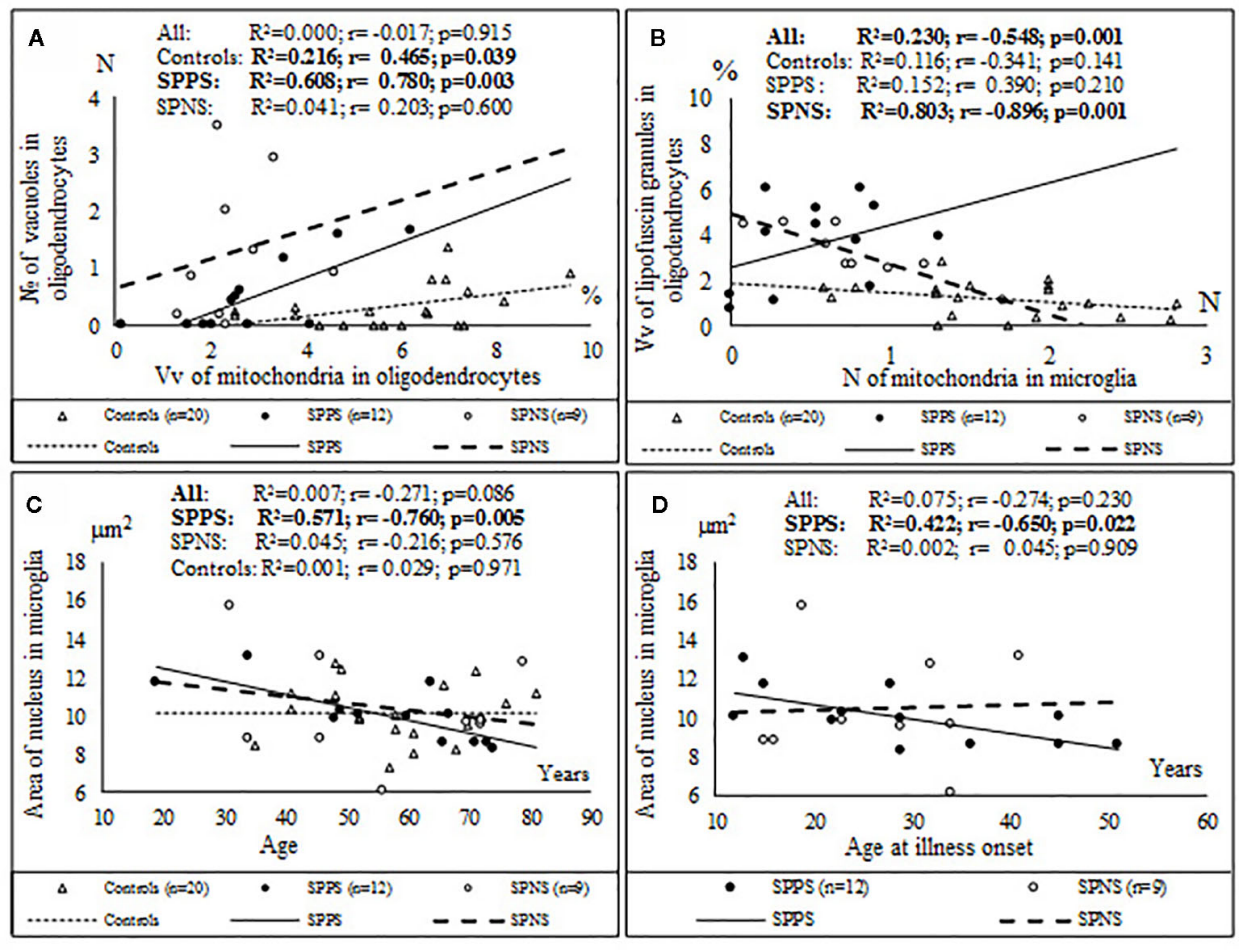
Positive significant correlations between Vv of mitochondria in oligodendrocytes and N of vacuoles in oligodendrocytes in the SPPS subgroup and in the control group **(A)**; negative significant correlations between N of mitochondria in microglia and Vv of lipofuscin granules in oligodendrocytes in the SPNS subgroup **(B)** and negative significant correlations between area of nucleus of microglia and age **(C)** and age at onset of disease **(D)** in the SPPS subgroup.

Correlations analysis demonstrated that only in the SPPS subgroup Vv and N of mitochondria in microglia were correlated positively with Vv (r = 0.79, p < 0.01) and N (r = 0.58, p < 0.05) of mitochondria in oligodendrocytes (**Figures 4A, B**). In this subgroup Vv of mitochondria in microglia was correlated positively with Vv (r = 0.77, p < 0.01) and N (=0.76, p < 0.01) of vacuoles in oligodendrocytes (**Figures 4C, D**). Besides, in the SPPS subgroup Vv and N of mitochondria was correlated negatively with N of vacuoles in microglia (r = -0,61, p < 0.05) (**Figures 4E, F**). Vv of mitochondria in oligodendrocytes was positively correlated with N of vacuoles in oligodendrocytes in the SPPS subgroup (r=0.78, p < 0.01) as well as in the control group (r = 0.47, p < 0.05) (**Figure 5A**). In the SPPS subgroup area of nucleus of microglia was correlated negatively with age (r = -0.76, p < 0.01) and age at onset of disease (r = -0.65, p < 0.05) (**Figures 5C, D**). In the SPNS subgroup N of mitochondria in microglia was correlated with Vv of lipofuscin granules in oligodendrocytes (r = -0.9, p < 0.01) (**Figure 5B**).

**Figure 6:**
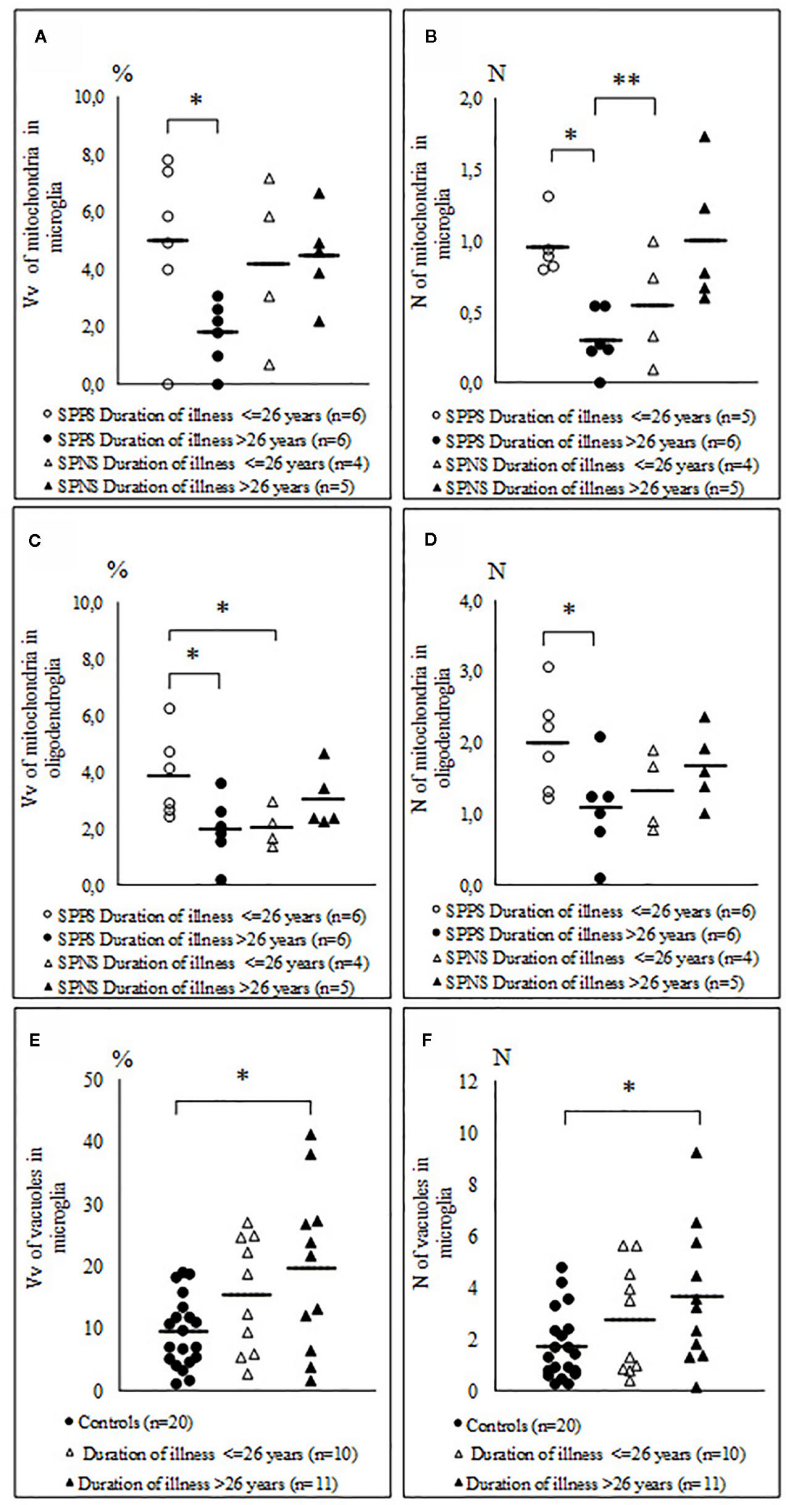
Individual and mean values for Vv and N of mitochondria in microglia **(A, B)**, in oligodendrocytes **(C, D)** in the SPPS and SPNS subgroups with shorter (< 26 years) and longer (> 26 years) duration of disease and Vv and N of vacuoles in microglia in the subgroups with shorter (< 26 years) and longer (> 26 years) duration of disease as compared to the control group **(E, F)**. *p < 0.05; **p < 0.01.

Comparison of the parameters measured in oligodendrocytes and microglia in the SPPS and the SPNS subgroups with shorter (< 26 years) and longer (> 26 years) duration of disease showed that in the SPPS subgroup Vv and N of mitochondria in both microglia and oligodendrocytes was significantly lower in the subgroup with longer duration of disease than in the subgroup with shorter duration of disease (p < 0.05) (**Figures 6A–D**). Vv and N of vacuoles in microglia was significantly higher in the subgroup with longer duration of disease as compared to the control group (p < 0.05) (**Figures 6E, F**).

**Figure 7:**
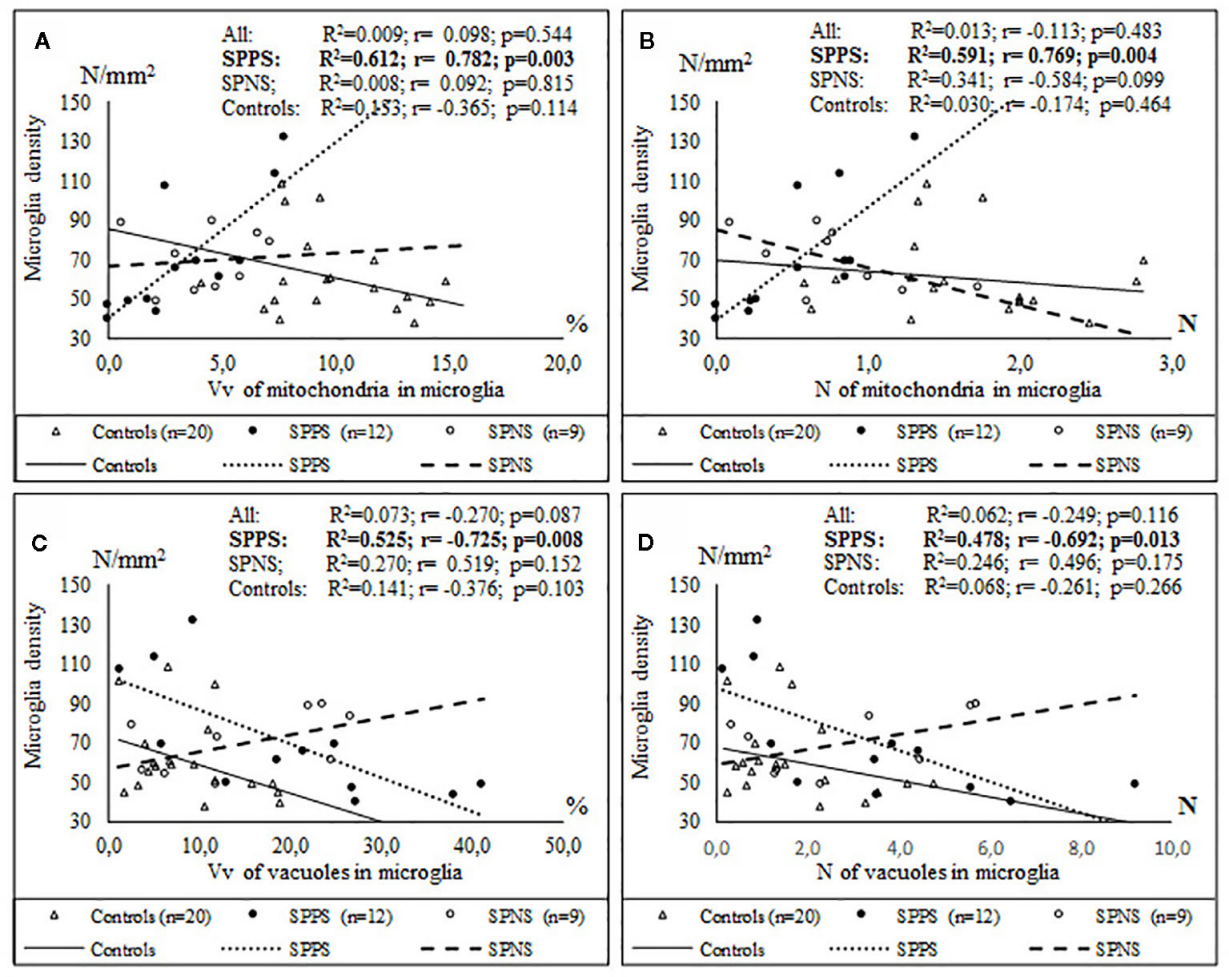
Positive significant correlations between Vv and N of mitochondria in microglia and microglial density **(A, B)** and negative significant correlations between Vv and N of vacuoles in microglia and microglial density **(C, D)** in the SPPS subgroup.

Only in the SPPS subgroup Vv and N of mitochondria in microglia were positively correlated with microglial density (r = 0.78, r = 0.77, p < 0.01). Alternatively, Vv and N of vacuoles in microglia were correlated negatively with microglial density (r = -0.72, p < 0.01; r = -0.69, p < 0.05) (**Figures 7C, D**). ANCOVA showed no significant effects of age, postmortem interval or gender on the parameters measured. No significant correlations between the parameters measured and postmortem interval, neuroleptic treatment were found.

## Discussion

The present study showed dystrophic alterations in microglia adjacent to oligodendrocytes and in oligodendrocytes adjacent to microglia in the schizophrenia group and in the SPPS and SPNS subgroups. The mean values of Vv and the N of mitochondria were significantly decreased while N of lipofuscin granules in microglia and oligodendrocytes were increased in the schizophrenia group as compared to the control group. However Vv of lipofuscin granules, Vv and N of vacuoles of endoplasmic reticulum in microglia were increased significantly only in the SPPS subgroup as compared to controls. These data provide evidence for more prominent alterations of microglia in the SPPS subgroup than in the SPNS subgroup. The important finding was the presence of positive correlations between Vv and N of mitochondria in microglia and Vv and N of mitochondria in oligodendrocytes as well as between Vv of mitochondria in microglia and Vv and N of vacuoles in oligodendrocytes only in the SPPS subgroup. These data point that dystrophic alterations in microglia are associated with the dystrophic alterations in oligodendrocytes in the SPPS subgroup. In the SPNS subgroup N of mitochondria in microglia correlated negatively with Vv of lipofuscin granules in oligodendrocytes. There were no significant correlations between all these parameters in the control group. Also, there were no significant effects of postmortem interval or gender, as well as no significant correlations between the parameters measured and postmortem interval, neuroleptic treatment. Neurodegenerative changes were excluded in the cases studied in neuropathological examination. Taken together, these data suggest that altered relationships between microglia and oligodendrocytes are associated with chronic schizophrenia and with deleterious effect of the disturbed metabolism in microglia on the oligodendrocyte metabolism in the SPPS subgroup in acute stage of disease, during relapse. Similar alterations of oligodendrocytes adjacent to microglia have been previously reported in the prefrontal white matter in chronic schizophrenia cases (21).

We found significant positive correlations between Vv and N of mitochondria in microglia and Vv and N of mitochondria in oligodendrocytes only in the SPPS subgroup. These data as well as the deficit of mitochondria in both microglia and in oligodendrocytes suggest that the damage and deficit of mitochondria in microglia and in oligodendrocytes may be crucial in the disturbance of microglial and oligodendrocyte metabolism in schizophrenia, especially in the SPPS subgroup, during acute state of disease. Mitochondria play a key role in cellular energy potential, calcium buffering, production of reactive oxygen species (ROS), they participate in regulation of apoptosis (42, 43). The deficits of mitochondria found in the present study is consistent with alterations in mitochondrial ultrastructure and number (13–15, 44), energy metabolism and oxidative stress, including the PFC (45–47). Defect of oxidative phosphorylation (reduced activity of complex IV) in the frontal cortex (48), altered expression of mitochondria-related genes including encoding respiratory chain components (49) have been reported in schizophrenia.

Flatow et al. (50) provide evidence for oxidative stress as a “potential biomarker in the pathophysiology and clinical course of schizophrenia”. Mitochondrial dysregulation may lead to oxidative stress and inflammation (51). Mitochondrial regulation of psychological stress reactivity systems has been recently reviewed (52). Postmortem studies have shown reduced levels of anti-oxidant glutathione in the PFC in chronic schizophrenia patients (53, 54). In response to oxidative stress damaged mitochondria might activate immuno-inflammatory pathways (55). ROS activation of transcription factors induced the expression of inflammatory genes (56). Also, postmortem studies demonstrated increased expression of immune-related genes in the PFC in schizophrenia (57). Thus, mitochondria abnormalities found in the present study are consistent with the alterations in mitochondrial energy metabolism, oxidative stress and neuroinflammation in schizophrenia, including the PFC. Mitochondria have contacts with endoplasmic reticulum (ER), and Ca^2+^ flux from the ER to mitochondria supports the Krebs cycle (43). Interestingly, some evidence suggests microglial intracellular Ca^2+^ signaling as a target of antipsychotic effects for the treatment of schizophrenia (58). Stressors disturb ER homeostasis, and the misfolded proteins may induce ER stress (43). In mammals, several viruses, invasive bacteria and parasites induce ER stress responses (59). ER stress and increases in ROS production activate autophagy to degrade stressed cellular organelles to restore homeostasis (43, 60). Mitochondrial autophagy, or mitophagy, has an important role in mitochondrial homeostasis, dysfunction and aging (61). Bernstein et al. (62) hypothesized that increased mitochondrial phagy (mitophagy) in oligodendrocytes might participate in white matter pathology in schizophrenia. Taken together, these data support a crucial role of damage and deficit of mitochondria in disturbed metabolism and in the dysfunction of microglia and adjacent oligodendrocytes in schizophrenia.

Our study indicated a significant increase in Vv and N of vacuoles only in the SPPS subgroup as compared to controls. Qualitative study showed that vacuoles are formed by enlarged cisterns of rough endoplasmic reticulum. Vv of vacuoles of ER and lipofuscin granules were significantly increased in microglia and in oligodendrocytes in the SPPS subgroup as compared to the control group. The ultrastructural marker of ER stress is a lumen dilation of rough ER (63, 64). Vacuoles in microglia present dilated cisterns of rough ER, and increased Vv and number of vacuoles found in our study only in the SPPS subgroup appear to reflect ER stress.

ER stress regulates differentiation, activation of immune cells and cytokine expression (65). ER stress can trigger inflammation associated with damaged mitochondria (66). An NLRP3- caspase-2-dependent mechanism is necessary to connect ER stress to mitochondria to induce inflammation (66). In patients with schizophrenia inflammation and oxidative stress may influence each other resulting cellular damage (67). Taken together, these data suggest that altered communication between mitochondria and ER stress in microglia (in the SPPS subgroup) might contribute to increased production of proinflammatory cytokines in acute state of schizophrenia. The deficit of mitochondria, a significant increase in Vv and N of vacuoles in microglia and negative correlations of N of vacuoles with Vv and N of mitochondria found in microglia in the SPPS subgroup suggest that microglia in the SPPS subgroup may be compensatory overactivated due to ER stress, because increased number of vacuoles coexists with the deficit of mitochondria.

Our study showed different signs of microglial activation in schizophrenia: the presence of activated microglia (**Figure 1B**), contacts between microglial cytoplasm and nucleus of oligodendrocytes in schizophrenia but rare in the controls; irregular contour of nuclei and numerous vacuoles in cytoplasm of many microglial cells; focal lysis of cytoplasm of oligodendrocytes located near the contact with microglia supposedly due to toxic microglia effect on oligodendrocytes.

The presence of continuum of microglial cells from activated to prominent dystrophy and apoptosis of microglia found in the present study suggest that prolonged activation of microglial cells in the course of the disease might make these cells exhaustive and degenerating in chronic schizophrenia patients and might decrease their protective potential for oligodendrocytes and for other cells as well as the plasticity of microglia and oligodendrocytes in schizophrenia. Degeneration of microglia has been found in the frontal and temporal cortex in schizophrenia (68). In our study the cytoplasm of both dystrophic dark and apoptotic microglia touched the nucleus of oligodendrocytes suggesting direct contacts, a sign of their activation. Similar “dark” microglia becomes numerous during chronic stress (64). Dark microglia is supposed to be much more active than the normal microglia (64). Thus, our data indicate that microglia from patients with schizophrenia is in broad spectrum of changes - from activated to degenerating.

We did not find the effects of neuroleptic medication on the parameters measured. Antipsychotics are known to inhibit microglial activation (69). Recently Du et al., (70) in the model of transient global cerebral ischemia showed an anti-depressant effect of minocycline by inhibiting microglia activation, promoting OPCs maturation and remyelination. Both typical and atypical neuroleptics decrease cytokines such as IL-2, IL-6 and TNF-alpha by inhibiting microglial activation (71–73). Kung et al. (74) have reported a reduction in the numbers of mitochondrial profiles per axon terminal in the drug-free schizophrenia patients as compared to either on-drug patients or to controls. Taken together, the data suggest that the deficit of mitochondria in both microglia and oligodendrocytes is related to schizophrenia.

Recently we have reported reduced oligodendrocyte density (-32%) in layer 5 of the PFC (Brodmann's area 10) in schizophrenia (12). In the present study we found dystrophic changes but no degeneration of oligodendrocytes in layer 5 of the PFC in schizophrenia. Thus, a prominent deficit of oligodendrocytes in layer 5 of the PFC cannot be explained by current oligodendrocyte degeneration, it may be the result of disrupted maturation and proliferation of oligodendrocyte progenitors in schizophrenia (75–78).

Oligodendrocytes and especially oligodendrocyte precursors are highly sensitive to inflammation, oxidative stress, hypoxia-ischemia, elevated glutamate levels (8). Redox regulation has a crucial role in myelination and white matter maturation in the PFC (79). In response to stress, oligodendrocytes produce immune mediators that modulate activation of microglial cells (67). Oxidative stress during late adolescence impairs oligodendrocyte precursors signal transduction processes and disrupts oligodendrocyte maturation (80). ER stress and mitochondrial dysfunction are involved in death of oligodendrocyte precursors by unconjugated bilirubin (81). The disruption of glutamatergic, redox, immune systems could be deleterious for oligodendrocyte differentiation and myelination (82). N-acetylcysteine, a precursor of endogenous antioxidant glutathione, diminish the cuprizone-induced behavioral changes and oligodendrocyte loss in male C57BL/7 mice due to its anti-inflammation actions (83). On the other hand, microglia contribute to maintenance of oligodendrocyte progenitors during adulthood and in adults (84). Microglia activation triggers apoptosis of oligodendrocyte precursors by HSP60 (85). Increased oxidative stress and oxidative DNA damage have been detected in nonremission patients with schizophrenia (86). Maas et al. (87) provides a hypothetic link between oxidative stress, hypomyelination of the PFC and cognitive symptoms in schizophrenia. Together these data provide evidence for the crucial role of oxidative stress in disturbed metabolism of oligodendrocytes and microglia in schizophrenia during brain development and in adult patients.

We found that in the SPPS subgroup in both oligodendrocytes and microglia Vv and N of mitochondria were decreased in the subgroup with longer duration of disease (>26 years) than in the subgroup with shorter duration of disease (<26 years). These parameters were positively correlated between microglia and oligodendrocytes in the SPPS subgroup. Also Vv and N of vacuoles in microglia were significantly increased in the subgroup with longer duration of illness as compared to controls (**Figure 6**). Besides, area of nucleus of microglial cells was correlated negatively significantly with age in the SPPS subgroup. Neuroinflammation has recently been reported in the PFC in elderly chronic schizophrenia patients (88). Taken together, these data suggest that microglia is involved in a progressive course of schizophrenia. The suggestion is in line with the results of neuroimaging studies that “progressive” brain changes might occur in schizophrenia, supposedly associated with episodes of acute relapse (89, 90).

In our study Vv an N of mitochondria in microglia were correlated positively with microglial density, and Vv and N of vacuoles in microglia were correlated negatively with microglial density in the SPPS subgroup, though microglial density did not differ from the control group (**Figure 7**). Microglia are actively renewed in brain maintained by a balance of proliferation and apoptosis (91). This balance is probably disturbed in acute state of schizophrenia. Taken together the dystrophic alterations of microglia and adjacent oligodendrocytes in schizophrenia found in the present study may explain at least in part the association of a high relapse rate with poorer outcomes, reduced plasticity and functional disability in patients with schizophrenia.

### Limitations

Our study has some limitations. We had to deal with a relatively small number of oligodendrocyte-microglial pairs. Many subjects in our study were older than 55 years old with long duration of disease (average 26 years). The effects of neuroleptic treatment were not determined. Two separate scales used at the time of autopsy were used for the estimation of predominantly positive and predominantly negative symptoms.

## Conclusion

Dysregulated microglial reactivity and microglia dystrophy in acute stage of schizophrenia might contribute to the dystrophy of oligodendrocytes and might be involved in progressive course and reduced brain plasticity in chronic schizophrenia patients. Mitochondria in microglia and oligodendrocytes may be a target for treatment strategy of schizophrenia.

## Data Availability Statement

The datasets generated for this study are available on request to the corresponding author.

## Ethics Statement

Consent for autopsy was obtained and approval for the study from the Ethics Committee of Mental Health Research Center was received.

## Author Contributions

NU designed the study, wrote, and revised the manuscript. OV performed morphometry and prepared the draft of the manuscript. VR performed statistical analysis. DO prepared and revised manuscript.

## Funding

The study was supported from the Federal Budget by Mental Health Research Center, Russian Federation (the theme N° 0508-2014-0066).

## Conflict of Interest

The authors declare that the research was conducted in the absence of any commercial or financial relationships that could be construed as a potential conflict of interest.
